# Evolutionary science as a method to facilitate higher level thinking and reasoning in medical training

**DOI:** 10.1093/emph/eow029

**Published:** 2016-10-15

**Authors:** Joseph L. Graves, Chris Reiber, Anna Thanukos, Magdalena Hurtado, Terry Wolpaw

**Affiliations:** 1 Joint School for Nanoscience & Nanoengineering, North Carolina A&T State University & UNC Greensboro, Greensboro, NC, USA; 2 Department of Anthropology, Binghamton University, PO Box 6000, Binghamton, NY 13902-6000, USA; 3 University of California Museum of Paleontology, Berkeley, CA 94720-4780, USA; 4 Department of Anthropology, Arizona State University, Tempe, AZ 85281, USA; 5 Penn State Health, Pennsylvania State University, Hershey, PA 17033, USA

**Keywords:** evolution, core concepts, blooms taxonomy, medical training

## Abstract

Evolutionary science is indispensable for understanding biological processes. Effective medical treatment must be anchored in sound biology. However, currently the insights available from evolutionary science are not adequately incorporated in either pre-medical or medical school curricula. To illuminate how evolution may be helpful in these areas, examples in which the insights of evolutionary science are already improving medical treatment and ways in which evolutionary reasoning can be practiced in the context of medicine are provided. To facilitate the learning of evolutionary principles, concepts derived from evolutionary science that medical students and professionals should understand are outlined. These concepts are designed to be authoritative and at the same time easily accessible for anyone with the general biological knowledge of a first-year medical student. Thus, we conclude that medical practice informed by evolutionary principles will be more effective and lead to better patient outcomes. Furthermore, it is argued that evolutionary medicine complements general medical training because it provides an additional means by which medical students can practice the critical thinking skills that will be important in their future practice. We argue that core concepts from evolutionary science have the potential to improve critical thinking and facilitate more effective learning in medical training.

## INTRODUCTION

Many leading scientific and medical bodies recognize the growing importance of evolutionary science to biology and medicine [1–3]. For example, for some time now physicians have realized that intra-tumor heterogeneity is a major factor in cancer treatment failure [4–6]. Recent strides are being made to understand this heterogeneity better by using methods developed in evolutionary phylogenomics [6]. Of course, the reason that this approach is essential to understanding tumor dynamics is because as tumors grow, they also evolve. As renegade cancer cell lineages evolve, free from the controls of normal cell division, clones expressing genetic elements that increase their replication relative to others will begin to dominate the tumor through the process of natural selection. Furthermore, if drug therapy is used to control the tumor growth, some clones will evolve resistance to the particular drugs being administered, with resistant clones reproducing at greater rates than non-resistant clones. This is a classic case of natural selection producing adaptations to a novel compound, as seen in the case of bacteria evolving resistance to antibiotics or HIV evolving resistance to azidothymidine. This is just one example of the many areas of medicine to which evolutionary reasoning is increasingly critical (e.g. atopic disease, senescence, genetic variation and drug response) in diagnosis, treatment planning and research.

Since the physicians of the future will be employing more applications of evolutionary science in their practical work, it is crucial that we begin to engage today’s medical students around these concepts. Unfortunately, evolution education in US lags behind that of other nations with advanced scientific research programs, and misconceptions concerning the core principles of evolutionary science are widespread among the American public [7, 8]. Yet despite the importance of evolution to medicine, the pre-medical curriculum and medical colleges continue to offer sparse coverage of evolution. One recent study surveyed curriculum deans of North American medical schools allowing them to rate their curriculum for coverage of 12 core concepts in evolution. Of those surveyed, 60 schools (39%) responded to the survey. The deans rated three evolutionary principles as most important: antibiotic resistance, environmental mismatch and somatic selection in cancer. Despite this, coverage of evolutionary principles lagged behind the perceived importance of them by on average 21% [9]. This study also compared its results to a previous study [10] and found that the range of principles covered had improved between 4 and 74%. The Association of American Medical Colleges has recognized the need for improving the education of undergraduates in evolution [11]. Thus, the Medical College Admission test now contains some items that test prospective students’ comprehension of core evolutionary principles. This is a start, but clearly a few items on an admission test are insufficient to motivate a shift toward greater attention to evolution in medical education.

Recognizing the growing number of physicians and scientists focused on evolutionary science’s promise for addressing health and disease as well as the under-preparedness of medical students in this area, in 2011 the National Science Foundation (NSF)-supported National Evolutionary Synthesis Center convened a working group to address the role of evolutionary biology in medicine and medical education. The group, led by physician Mark Schwartz and evolutionary biologist Peter Ellison, consists of physicians, researchers in the fields of evolutionary medicine and public health and experts in evolution education. The group’s advisory board included the President of the Institute of Medicine and the Deans of two prestigious medical schools (see Appendix A for group members). In addition to recommending that more attention be paid in the medical curriculum to integrating core concepts of evolution into appropriate courses and subject areas, the group identified a set of crucial evolutionary concepts that should be included in all medical curricula. These concepts follow closely those recommended in previous analyses, and some examples of these concepts are presented in Table 1 [12].

**Table 1.** Exemplars of evolutionary concepts, learning objectives and competencies relevant to medicine

Concept	Learning objective	Competency
Adaptation/adaptive	Explain what is meant by adaptation and how adaptations are shaped by natural selection.	Students should be able to explain specific examples of adaptation and how they may impact specific diseases.
Examples in text: antimicrobial resistance; sickle-cell anemia; skin color variation		
Hygiene hypothesis	Explain the hygiene hypothesis.	Students should be able to explain how the hygiene hypothesis is applied to atopic disease.
Example in text: allergy prevalence in city v. country children		
Life history theory (life history evolution)	Explain how life histories evolve.	Students should be able to explain how life history theory accounts for aging.
Example in text: senescence (aging)		
Microbiome	Describe the human microbiome.	Students should be able to explain how variations in the human microbiome may be associated with specific diseases.
Examples in text: bacteria/parasitic worms and atopic disease; microbiome and metabolic disease		
Mismatch	Explain evolutionary mismatch.	Students should be able to explain how evolutionary mismatches may contribute to specific diseases.
Examples in text: novel (nano) materials; heart, cancer and metabolic disease pandemic in Western societies		
Natural selection	Define natural selection.	Students should be able to explain how natural selection molds the characteristics of a given species, including attributes of that species relevant to disease.
Examples in text: intra-tumor heterogeneity; antimicrobial resistance; sickle-cell anemia; skin color variation		
Race (biological and socially defined)	Define biological and socially defined race.	Students should be able to explain the difference between biological race categories and socially defined categories. Specifically students should understand the relevance of this distinction to addressing health disparities.
Examples in text: sickle-cell anemia; olanzapine response variants; skin color variation; pain tolerance myths		
Trade-offs	Define an evolutionary trade-off.	Students should be able to explain why the existence of trade-offs means that no bodily system can be perfect.
Examples in text: senescence (aging); intermediate loads of parasite levels		

Definitions are provided in the understanding evolution website glossary, http://evolution.berkeley.edu/evolibrary/glossary/glossary.php (13 October 2016, date last accessed).

## ESSENTIAL LEARNING OBJECTIVES

These evolutionary science concepts can be thought of as a series of learning objectives, which, if mastered by a student, entail a greater understanding of both evolutionary reasoning and content knowledge relevant to medical practice. Given the inadequate training in evolutionary science that exists in our present undergraduate education system [8] and the depth to which evolutionary biology pervades medical phenomena, memorizing the definitions of important evolutionary science terms will not be enough for modern medical students. This approach amounts to retrieving information from memory without necessarily being able to use the knowledge in any way and represents the simplest sort of learning (Level 1) according to Bloom’s Revised Taxonomy [13]. This taxonomy is a widely used tool in teaching, including in medical education. For example, it has been recently used to measure cognitive processing and judgments of knowledge in medical students, measuring the design and evaluation of assessment tools in the anatomical sciences, the impact of flipped classrooms in anatomy instruction and commitment to change in clinical practice [14–17]. The popularity of this method is that it allows assessment of student learning that separates superficial from deep learning [17]. It accomplishes this by providing carefully developed definitions of six major categories in the cognitive domain: remembering, understanding, applying, analysing, evaluating and creating.

Here, we propose that Bloom’s taxonomy may also be employed to accomplish deep learning of evolutionary principles for medical students. For example, natural selection can be defined as differential survival or reproduction of individuals that result in changes in the frequency of heritable traits in a population. These individuals may represent different genotypes leading to changes in allele frequencies of the population. In some cases, the individuals may represent different phenotypes which, while identical in genetic sequence, are different phenotypically due to epigenetic changes (e.g. DNA methylation), or chance events that occurred during development and which are passed down through generations. There are four conditions required for evolution by natural selection to occur: variation, heredity, differential reproduction and time. Level 1 of Bloom’s taxonomy (Table 2) simply requires a student to remember this definition.

**Table 2.** A Bloom’s taxonomy of natural selection

Level	Action	Example
1	Remembering	Students know and can recite the definition of natural selection.
2	Understanding	Students understand that positive natural selection increases the frequency of variants that improve reproduction and survivorship in a specific environment, such as in the case of antibiotic resistance in bacteria.
3	Applying	Students can apply the concept of natural selection to a new situation. For example, after learning about progeria, students should be able to predict that the expected frequencies of deleterious variants responsible for the genetic disease will be equivalent to their mutation rate since persons with progeria rarely reproduce.
4	Analysing	Students will be able to analyse how different models of natural selection would account for observations. For example, a correct model of natural selection can account for the frequency of diseases such as sickle-cell anemia (due to heterozygote superiority) and can explain the prevalence of alleles that provoke negative drug interactions in some patients but not others.
5	Evaluating	Students can evaluate specific evolutionary hypotheses to determine which ideas may have traction with regards to improving treatment and overall patient outcomes; e.g. how good is the evidence supporting the hygiene hypothesis? What does the hypothesis predict? What observations are not supported? For example, students could evaluate evidence for and against the hygiene hypothesis.
6.	Creating	Students can now use evolutionary science to create their own hypotheses relevant to improving treatment and patient outcomes. Students would be able to formulate evolutionarily informed hypotheses to address the spread of complex illnesses due to globalization or increases in such diseases due to the introduction of novel substances in the environment or diet.

Level 2 (understanding) requires the student to understand how natural selection works and how this might impact medicine. Natural selection is the means by which organisms acquire adaptations (that is, characteristics that improve an organism’s differential survival and reproduction) in specific environments. The student will now need to grasp how natural selection molds (and in some cases fails to mold) not just the features of humans but also the features of the organisms that contribute to their health and disease. A truly motivated student might even grasp more complicated concepts, such as how natural selection plays a role in shaping the life history features of humans, including senescence—a fundamental issue in medical practice. In addition, they would be able to comprehend how positive natural selection for antibiotic resistance would spread resistance alleles through pathogen populations and how positive natural selection would result in drug resistant cell lineages spreading through tumors.

At Level 3 (applying) students are expected to be able to apply procedural knowledge related to natural selection to a medical issue. For example, they should be able to calculate the differential reproductive success of genotypes given sufficient information, such as the survival probabilities of the genotypes and their fecundities. Similarly, if a student at Level 3 were given the age-specific survivorship and fecundity of patients with a particular genetic trait (such as progeria), he or she should be able to demonstrate that natural selection would reduce the frequency of such an allele to a very low level in any population. Another very good example of applying natural selection to a problem of medical significance is antimicrobial resistance. Students should be able to realize that the way in which antimicrobials are applied to treat a patient’s infection will have profound impacts on the ability of the microbe to evolve resistance. For example, will there be a difference between treating the infection until the patient feels better (and their own immunity can handle the infection) or should treatment be continued for a fixed period with the goal of using the antimicrobial to completely eradicate the infection?

Level 4 (analysing) requires the student to analyse natural selection; such an analysis would lead the student to the logical implications that natural selection has for the field of medicine. Because this level is particularly important for medical practice, we will provide an illustrative example. The first cases of sickle-cell anemia were described in the latter portion of the 19th century [18]. Emmel found the trait in the father of one of the first reported sickle-cell anemia cases, which suggested a genetic basis to the disease [19]. By the 1940s, it was realized that sickle-cell anemia was widespread in tropical-equatorial Africa, with the severe form more rare than the moderate form [20, 21]. This led researchers to conclude that sickle-cell anemia was inherited as an autosomal Mendelian dominant trait [18]. Yet at the same time, the symptoms of sickle-cell anemia were quite severe. Doctors observed a very high death rate among young children with the disease [19]. With these facts at hand, how was it that physicians arrived at the conclusion that sickle anemia was caused by an autosomal dominant trait? Clearly, they did not have the level of understanding of natural selection that would be needed to analyse the logical effect of natural selection on a highly deleterious autosomal dominant trait. If they had been analysing the situation in light of natural selection, they would have asked the obvious question: if this trait is an autosomal dominant, how can it be so frequent? Natural selection would cause an autosomal dominant trait with such drastic effects on survival to be eliminated from the population rapidly. Indeed, the evolutionary theory needed to understand this situation had already been authored by Haldane and Fisher [22, 23]. If physicians had applied such reasoning to this case, they would likely have recognized that they were, instead, dealing with a recessive trait and a case of heterozygote advantage. Sickle-cell anemia is now recognized as one of the best-documented cases of evolution in action in humans both as heterozygote advantage and an anti-malarial adaptation [24].

The medical implications of having a false model of the genetics of sickle-cell anemia (resulting from the failure to analyse the disease through an evolutionary lens) are important. If the trait is understood to be dominant, a physician providing genetic counseling to a patient displaying the disease would claim that, on average, 50% of the patient’s children would inherit the disease. On the other hand, if the trait is understood to be a case of heterozygote advantage, the patient’s children are only expected to display the disease condition if the patient’s partner’s family carries the sickle-cell allele.

Another well-documented example of how analysing the process of natural selection can be relevant to medical practice involves human genetic variation. Based on evolutionary concepts such as natural selection, students should expect that patients carry medically relevant genetic variants and that these variants are unlikely to conform to socially defined concepts of ‘race’. For example, past natural selection impacts how patients respond to the drugs used to treat their illnesses. Numerous polymorphisms have been discovered that are strongly linked to the ability of patients to tolerate the antipsychotic drug olanzapine [25]. In one study, 63 persons of European ancestry were examined, and the following loci with polymorphisms associated with negative response to olanzapine were found: CYP2C9 (17.5% carried the allele with risk of negative response), TPMT (6.3% genotypes with risk), UGTIA1 (50.8% carried the risk allele), MDR1 (22.2% genotype at risk) and 5-HTR2A (66.7% carried the risk allele) [25]. An important learning objective for medical students at Level 4 of Bloom’s taxonomy for natural selection is to be able to analyse how the sorts of genetic variation that human populations maintain may have been influenced by episodes of natural selection (or other evolutionary processes such as gene flow or genetic drift) in the past, and how this may relate to medical interventions today. Furthermore, the student with this level of understanding of natural selection (and other mechanisms of evolution) would recognize that human genetic variation does not match socially defined categories of race or ethnicity but is influenced by the evolutionary history of individual human populations. This is because socially defined races are discordant with both the physical and genetic variation observed in our species. For example, natural selection that influenced the frequency of alleles associated with skin color variation, did not at the same time determine the frequency of alleles associated with any specific disease predisposition or for height [26]. Thus, while we would expect there to be alleles of medical relevance that are differentiated by ancestral geographical factors, we would not expect there to be drugs with impacts that are race-specific as has been frequently claimed [27, 28]. This does not mean that there are not differences in the how individuals or populations respond to drugs; it simply means that these responses do not correspond to socially defined notions of race. A particularly troubling example of this ongoing misconception was revealed by the fact that in a recent study one-half of the medical students surveyed harbored false beliefs concerning biological differences between socially defined racial groups. In conjunction with these false beliefs they rated the pain of ‘black’ lower than the pain of ‘white’ patients and as a result made inappropriate treatment recommendations for the ‘black’ patients [29]. The widespread lack of understanding of this issue amongst medical practitioners and biomedical research scientists is an ongoing problem that training in evolutionary reasoning is uniquely suited to address [30].When applied to natural selection, Levels 5 (evaluating) and 6 (creating) of Bloom’s taxonomy may also be viewed as helpful for medical practitioners and critical for medical researchers. Level 5 means that a student can evaluate specific hypotheses and research related to the impact of natural selection upon medicine. The hygiene hypothesis is one example of a current idea from the field of evolutionary science that has gained some traction lately in the treatment of allergic and autoimmune disease [31, 32]. This hypothesis seeks to explain the recent rise in autoimmune disorders—e.g. it has been estimated that at present at least 40% of the populations of USA and Europe suffer from one or more types of allergy and that the prevalence of allergies in Western industrialized societies has doubled in the last 15 years [33, 34]. The hygiene hypothesis is a specific variant of the ‘novel environment’ idea. It argues that some of our body systems evolved to function in the presence of infection by microorganisms and parasitic worms, to which we were continuously exposed throughout our evolutionary history. According to the hygiene hypothesis, in a modern industrialized Western environment where worm and parasite exposure is infrequent, these physiological processes malfunction resulting in the rising prevalence of atopic disorders and autoimmune diseases [34].Evaluation of the hygiene hypothesis would be improved by addressing additional questions that are inspired by other evolutionary concepts. First, what trade-offs are associated with infection by microorganisms and parasitic worms? We have some data relevant to evaluating the downside to parasitic microorganism and intestinal worm (roundworm and flatworm) infection. In 1914, the Rockefeller Public Service Commission found that 39% of southern school children (European-American) were infected with the roundworm (*Necator americanus*, called hookworm) [35]. Individuals with severe hookworm were shown to have extreme lassitude (mental and physical) due to severe anemia. It is not hard to demonstrate that these infections cause significant mortality [36]. Therefore, natural selection would select against *N.americanus* susceptibility and for resistance to such a parasite.

Although the negative fitness impacts of parasitic infection are relatively simple to demonstrate, the more significant task is to demonstrate that low to intermediate levels of the antigens of specific microorganisms and parasitic worms have a beneficial effect on health. Specifically, the hygiene hypothesis argues that the differences we see between non-sufferers and those suffering from allergic, atopic and autoimmune disease are primarily caused by shifts from our ancestral (agricultural) to modern (industrial) environments. The student with Level 5 understanding will know how to determine if the increased prevalence of these diseases could be due to a ‘mismatch’ of humans and industrialized environments with few opportunities for infection by worms—and if intermediate levels of worm infection offer some protection from these disorders.

Several lines of evidence are consistent with the idea that intermediate levels of microorganism and parasite infection can be beneficial for health. A meta-analysis of papers concerning helminth infection and metabolic syndrome found that individuals with a previous or current helminth were 50% less likely to have an endpoint metabolic dysfunction comparted to uninfected individuals [37]. In addition, there is evidence that the human intestinal microbiomes (here including parasitic worms) influence who develops allergy, atopy and autoimmune disease and who does not. For example, one study of school age children in the tropics found a reduced risk of atopy due to moderate infections with helminths [38]. Infants who had or were developing atopic disease where shown to have less diverse microflora than those without the disease [39, 40]. Furthermore, several studies have shown that children in farm environments have less allergic disease compared with those raised in urban environments, including Amish children in northern Indiana [41], children living in Alpine farm environments [42] and an Austrian study that showed there was more hay fever and asthma in children living in city versus farm environments, where a more diverse microbiome is likely to be acquired [43]. In addition, Azad *et al.* utilized a natural experiment to show that microbial diversity increased in the guts of children exposed to pets, which simultaneously resulted in lower levels of atopy and allergic disease relative to children with lower levels of diversity in their gut flora [44], and in another experiment, patients who were inoculated with eggs from *Trichuris suis*, a flatworm that does not cause disease, saw a significant reduction in Crohn’s disease [45]. The results of this study are additionally supported by an unmatched case-control study in South Africa that showed that helminth infection was protective against both Crohn’s disease and ulcerative colitis [46]. Growing evidence is confirming the idea that the gut microbiome appears to be intimately tied to immune response; hence, when it is dis-regulated we observe allergy, atopy and autoimmune disease [33, 47].

The identification and evaluation of the sorts of evidence described earlier, relevant to the hygiene hypothesis, should be achievable by a student with Level 5 competence with natural selection. These data would allow the student to evaluate the utility of this hypothesis (and its variants: the early immune challenge and the old friends hypothesis). In addition, the student should be able to evaluate data challenging the hypothesis. For example, there is evidence of an inverse relationship between some infections (such as Hepatitis A) and microbiome diversity. This result (which supports the importance of microbiome diversity as a protection against infectious disease) has been reproduced in some populations but not in others; and in fact, there is still a great deal of inconsistency in the results of human intervention studies utilizing pre- and probiotics to alleviate allergy [33, 47].

Finally, Level 6 of Bloom’s taxonomy requires that students be able to formulate and test their own hypotheses regarding the impact of natural selection on medicine. We argue that producing students with this level of understanding will have great benefit for biomedical research as well as clinical practice. Level 6 involves students creating new ideas and products. With such understanding, students can suggest new ways of approaching health and disease. Currently, many of these insights are provided by evolutionary biologists with an interest in medicine. However, with more thorough grounding in evolutionary concepts and reasoning skills, the doctors and medical researchers of tomorrow will be able to develop new hypotheses for investigation that are more fully informed by an understanding of medical phenomena. For example, the widespread adoption of evolutionary thinking in medicine is likely to have a profound influence on how to approach preventive medicine. Indeed, many evolutionary scientists today are arguing that the main cause of the dramatic increase in the prevalence of major chronic diseases (heart disease, cancer and diabetes) experienced by Western industrial populations is the mismatch between the environments in which natural selection acted to mold our current physiology (pre-agricultural) and our current post-industrial existence [48–52]. Due to globalization, the Western world has been exporting its unhealthy lifestyle around the world. Thus, we are now beginning to see dramatic increases in diseases such as diabetes in the Asian-Pacific rim [51]. In addition, it is likely that evolutionary mismatches are also contributing to the rapid increase in mental health disorders we are observing in Western industrialized societies [52]. Again, an evolutionary perspective would allow us to predict that we will see increasing prevalence of mental illness concurrent with the exportation of the Western industrial lifestyle and the Western agricultural diet to cultures that hitherto had been less exposed to these mismatches. A simple, clear and testable evolutionary hypothesis would be that adopting diets and lifestyles that are more consistent with our evolutionary history will lessen the prevalence and severity of such diseases. Doctors and medical researchers with a strong background in evolutionary concepts will be able to take these lines of research in new, more concrete and likely unanticipated, directions.

## CONCLUSION

Clearly, the ramifications of natural selection for medical practice and research are wide-ranging. To be prepared to practice medicine in the 21st century, medical students need to master the concept of natural selection, as well as other evolutionary concepts fundamental to medicine (such as those illustrated in Table 1). The application of Bloom’s taxonomy to mastering the fundamental evolutionary concept of natural selection is summarized in Table 2. The importance of this sort of basic evolutionary background for medical practice is likely to increase over time. A case that well-illustrates this point is the continuing advance of genetic technologies. Next generation sequencing developments are reducing cost and increasing speeds at rates in excess of those predicted by Moore’s Law [53]. This will mean even greater amounts and sophistication of the genetic data available for individual patients. Yet even with this increase in genomic information, there are signs of trouble with regard to the gaps in biomedical researchers’ training [54]. In addition, evolutionary medicine is a vibrant and growing field of inquiry. A June 2014 query on the Entrez Pubmed search engine returned 7025 citations under the term ‘evolutionary medicine’—less than one-half that returned from the term ‘personalized medicine’ at 15 207; 2 years later those same terms returned 10 354 citations under the term ‘evolutionary medicine’ and the term ‘personalized medicine’ had grown to 29 589 citations. This indicated that the difference between publications focusing in these two areas has grown over the last 2 years. Despite this difference in emphasis in the biomedical literature, we would argue that the relevance of many of evolutionary medicine’s tenets such as ‘evolutionary mismatch’ is actually increasing. This is due to increasing globalization and the export of the Western lifestyle around the world, and the rate at which Western societies are accelerating away from the conditions under which our species evolved. These forms of environmental change will bring about new health challenges that will be best addressed with an evolutionary perspective. For example, the 21st century has seen tremendous strides in technological development. One of the newest of these, nanotechnology will widely introduce novel materials (nanoparticles and nanomaterials) into the biosphere. Nanoparticles may result from natural processes (such as fires), be industrial byproducts (such as those produced in diesel exhaust) or be specifically engineered for their nanoscale properties. Humans have been exposed to naturally produced nanoparticles for some time; however, the last decade has seen a massive increase in and revolution in the types of engineered nanomaterials [55]. These new, engineered nanoparticles include particulates that have never been studied and other particulates that have been previously only been studied as components of mixtures [56]. This drastic growth in the production of nanomaterials will become an immediate concern for medical toxicology, since at present little research is being directed at nanosafety [57]. Evolutionary theory alerts us to the possibility that these new compounds could be highly toxic to living organisms. In addition, given that nanoparticles are already in use as biocides against bacteria, evolutionary theory suggests that there is a strong potential for the rapid evolution of nanoparticle resistance and the spread of this throughout the microbiome [58]. Other industrial processes will have significant impacts for medicine in the 21st century and have inherent ties to evolutionary biology. Anthropogenic climate change is likely to increase the rate at which novel infectious diseases enter the human population. This is because as the climate warms, the vector organisms that transmit many dangerous human infections will increase their range [59, 60]. This already is being proposed as a causal factor in the spread of the Zika virus [61]. In addition, globalization has increased the capacity of these organisms to be transported to new habitats [62]. Evolutionary theory can help us understand, analyse and sometimes even predict or alter the trajectory of emerging infectious disease.

We argue that the future of medical research and practice will increasingly require an evolutionary perspective to address the new health concerns of the 21st century. These will include chronic disease, mental health, as well as other issues such as emerging pathogens. The ability of physicians and biomedical researchers to link ultimate evolutionary explanations for disease to their proximate mechanisms shall become increasingly important. Therefore the sooner we revise medical preparation to integrate evolutionary perspectives, the better primed we will be to address the medical challenges of the 21st century.

## FUNDING

This work was made possible via a working group convened at the National Evolutionary Synthesis Center (NESCent). NESCent was supported by National Science Foundation (https://www.nsf.gov/) grant #EF-0905606.

**Conflict of interest:** None declared.

## Appendix A. Members of the infusing evolution into the Medical Curriculum Working Group

Working Group

Dr Mark Schwartz, MD, PI, Internal Medicine & Medical Education, NYU School of Medicine.

Peter Ellison, PhD, Co-PI, Anthropology & Evolutionary Biology, Harvard University.

William Aird, Vascular Medicine, Harvard University School of Medicine.

Athena Aktipis, PhD, Psychology and Evolution, Arizona State University & University of California, San Francisco.

Cynthia, Beall, PhD, Anthropology, Case Western Reserve University.

Gillian Bentley, PhD, Anthropology, Durham University.

Peter Gluckman, MD, PhD, Pediatrics, Perinatal Biology, University of Auckland, Liggins Institute.

Joseph L. Graves Jr, Evolutionary Biology, Joint School of Nanosciences & Nanoengineering, North Carolina A&T State University.

Brandon Hidaka, BS, Medicine, University of Kansas Medical Center.

Magdalena Hurtado, PhD, Human Evolution and Social Change, Arizona State University.

Barbara Natterson, MD, Cardiology, University of California, Los Angeles.

Randolph Nesse, MD, PhD, Psychiatry & Evolutionary Medicine, University of Michigan.

Chris Reiber, PhD, MPH, Biomedical Anthropology & Epidemiology, Binghamton University.

Stephen Stearns, PhD, Evolutionary Biology, Yale University.

Anna Thanukos, Evolution Education and Public Outreach, University of California Museum of Paleontology.

Terry Wolpaw, MD, Internal Medicine & Medical Education, Pennsylvania State University.

Advisory Board

Harvey Fineberg, MD, PhD, Chair, Health Policy, Institute of Medicine.

Robert J. Alpern, MD, Nephrology, Yale University School of Medicine.

David, Asai, PhD, Undergraduate Science Education, Howard Hughes Medical Institute.

Carol Aschenbrener, MD, Pathology, AMC, Culture & Education, Association of American Medical Colleges.

Jeffrey Flier, MD, Endocrinology, Harvard Medical School.

Jay Labov, PhD, Biology, Education, National Academy of Sciences, National Research Council.

Gilbert S. Omenn, MD, PhD, Computational Medicine & Bioinformatics, Internal Medicine, Human Genetics, and Public Health, University of Michigan.
